# Five novel *PRNP* gene polymorphisms and their potential effect on Scrapie susceptibility in three native Ethiopian sheep breeds

**DOI:** 10.1186/s12917-020-02336-0

**Published:** 2020-04-29

**Authors:** Eden Yitna Teferedegn, Yalcin Yaman, Cemal Un

**Affiliations:** 1 Ege University, Department of Biology, Molecular Biology Division, Izmir, Turkey; 2 Department of Biometry and Genetics, Bandirma Sheep Research Institute, Bandirma, Balikesir, Turkey

**Keywords:** Ethiopian sheep, Novel variations, Polymorphism, Prion gene, Scrapie susceptibility

## Abstract

**Background:**

Classical scrapie susceptibility in sheep has been linked to three polymorphisms at codon 136, 154, and 171 in the prion protein gene (*PRNP)* whereas atypical scrapie susceptibility is related to polymorphisms at codon 141. Many other variants over the length of the *PRNP* have been reported. Some of the variants may play crucial roles in fighting against the emergence of a new form of scrapie disease. Scrapie surveillance, scrapie associated genotyping and *PRNP* characterization studies have been conducted across the globe. However, such in-depth studies have never addressed the African continent’s sheep breeds. Therefore, genotyping native Ethiopian sheep breed’s *PRNP* gene has socioeconomic and scientific merits. This study aimed to identify *PRNP* variants in three native Ethiopian sheep breeds and their potential effect on scrapie susceptibility.

**Results:**

Five novel variants were identified in the *PRNP* gene of three native Ethiopian sheep breeds. Four non-synonymous heterozygous substitutions i.e. H99Q (CAC-- > CAA), H99L (CAC-- > CTA), A116E (GCA-- > GAA), A116T (GCA-- > ACA), and one synonymous N103 N (AAC-- > AAT) were detected. In addition to the novel variants, polymorphisms at codon 126,127,138,142,146,231, and 237 were also identified. The haplotype ARR was observed in Menz and Afar breeds at frequencies of 0.02 and 0.05 respectively. Neither ARR/ARR nor VRQ/VRQ genotypes were identified in the population under study.

**Conclusion:**

Two of the novel variants at codon 99 and 103 that are placed closer to the proteinase K cleavage site and the variant at codon 116 in the palindrome region along with variants at codon 127 in glycine repeat domain may influence the conformational flexibility of prion protein. The rarity of ARR haplotype and the abundance of 141 L variant demonstrated that the present study population was less resistant to classical scrapie and less predisposed to genotype associated atypical scrapie. This study provides a valuable dataset that can be potentially integrated into selective breeding strategies during interbreeding, crossbreeding and help to take precautionary measures against scrapie.

## Background

Prion diseases are a collective name for infectious neurodegenerative diseases caused by the misfolding of prion protein [1] .The infectious form of prion protein (PrP^Sc^) has different structural dynamics than cellular prion (PrP^C^). Specific motifs of prion protein were identified in relation to the conversion of PrP^C^ to PrP^Sc^ [2]. Amino acids at codons 141 and 154 were reported to be correlated to different forms of scrapie through altering the conformational flexibility of the prion protein [3].Hence; variants of such kind may play a crucial role in fighting against the emergence of a new form of scrapie.

Though the exact underlined disease mechanism is not yet known, scrapie transmission and susceptibility have been linked to the host genetics. Previous studies identified three polymorphic codons (136A/V, 154R/H, and171Q/R/H) in sheep *PRNP* (prion protein gene) that are related to scrapie resistance/susceptibility status [4–7]. Based on National Scrapie Plan for Great Britain, there are 15 known genotypes and 5 types/ groups that are associated to resistance or susceptibility to scrapie. Accordingly, ARR/ARR is the most resistant genotype that is classified under type/group 1, ARR/XXX except VRQ is categorized under type 2, the combination of ARQ, AHQ and ARH alleles have little resistance to scrapie and classified under group 3, ARR/VRQ is genetically susceptible genotype that is classified under type 4 and VRQ/XXX except ARR is classified under highly susceptible group [8]. In atypical scrapie case, susceptibility is higher in individuals with AHQ, AHQ/ARQ and ARR genotypes along with homozygosity for phenylalanine at codon 141 [9]. An earlier study linked AC_151_RQ genotype to prolonged incubation period after scrapie exposure [7]. Similarly, several studies reported variants such as G126A, G126G, G127G, G127V, G127A, and S138S [10–12] in sheep prion protein with or without direct effect to scrapie susceptibility.

In the past several years there were efforts to genetically characterize local breeds of many countries in identifying new variants and determining scrapie resistance/susceptible haplotypes. Based on the findings, measures were taken to control and reduce transmission of transmissible spongiform encephalopathy horizontally and vertically [13–15]. However, such type of extensive studies have never been conducted in Sub-Saharan African countries, such as Ethiopia, where livestock is the main economic source and a large proportion of the population depends on livestock products. On the other hand, the ever-increasing animal product demand enhances crossbreeding programs for the last few decades through the importation of exotic animals and the distribution of crossbred F1 in different parts of Ethiopia. Such practices are the potential factors in changing the genetic structure of the population and may introduce new disease susceptible genotypes [16, 17]. Hence, genotyping native Ethiopian sheep breeds *PRNP* gene has public health, economic and scientific merits [18]. This study aimed to identify *PRNP* variants in three native Ethiopian sheep breeds and their potential effect on scrapie susceptibility.

## Results

In the present study, five novel *PRNP* variants were detected in the three native Ethiopian sheep breeds. Heterozygosity at nucleotide 296 and 297 resulted in two variants at codon 99 i.e. H99Q (CAC-- > CAA) and H99L (CAC-- > CTA). These variants were observed at < 0.13 frequency in the population under study Table 1. Similarly, two new substitutions at the previously reported polymorphic site i.e. A116T (GCA-- > ACA) and A116E (GCA-- > GAA) were identified. The novel variants at codon 116 were not detected in Afar breed. The A116E variant was observed at 0.03 and 0.10 frequencies in Menz and Washera breeds respectively. A116T was also observed in 8% of Menz and Washera breeds Table 1. A synonymous substitution i.e. N103 N (AAC-- > AAT) at the previously described polymorphic site was also identified. This synonymous variant was not detected in Afar breed.

**Table 1:** Frequencies of Novel and additional variants in Menz, Washera and Afar sheep breeds of Ethiopia

Variants	MENZ	WASHERA	AFAR
	(*N* = 35)	(*N* = 39)	(*N* = 23)
99H	0.94	0.87	0.83
H99Q* (CAC--CAA)	0.03	0.08	0.04
H99L*(CAC-- > CTA)	0.03	0.05	0.13
103 N	0.91	0.69	1
N103 N*(AAC--AAT)	0.09	0.31	–
116A	0.94	0.85	1
juA116E*(GCA--GAA)	0.03	0.1	–
A116T*(GCA--ACA)	0.03	0.05	–
126G	0.91	0.62	0.78
G126A(GGG--GCG)	0.06	0.08	–
G126A*(GGG--GCG)	0.03	0.3	0.22
127G	0.6	0.59	0.88
G127V* (GGG-- > GTT)	0.23	0.31	0.04
G127A* (GGG-- > GCG)	0.11	0.01	–
G127G(GGG-- > GGT)	0.03	0.03	–
G127A* (GGG-- > GCC)	0.03	0.03	0.04
G127V* (GGG-- > GTC)	–	0.03	0.04
138S	0.94	0.95	0.74
S138S(AGC-- > AGT)	0.03	0.05	0.17
S138S*(AGC-- > AGT)	0.03	–	0.09
I142	1	0.77	23
I142T*(ATA-- > ACA)	–	0.23	–
N146	0.91	0.94	0.79
N146S(AAT-- > AGT)	–	0.03	0.08
N146S*(AAT-- > AGT)	0.09	0.03	0.13
R231	0.4	0.18	0.39
R231R*(CGG-- > AGG)	0.37	0.33	0.39
R231R(CGG-- > AGG)	0.23	0.49	0.22
L237	0.69	0.13	0.35
L237L(CTC-- > CTG)	0.08	0.49	0.26
L237L* (CTC-- > CTG).	0.23	0.38	0.39

*Hetrozygous

**Fig. 1:**
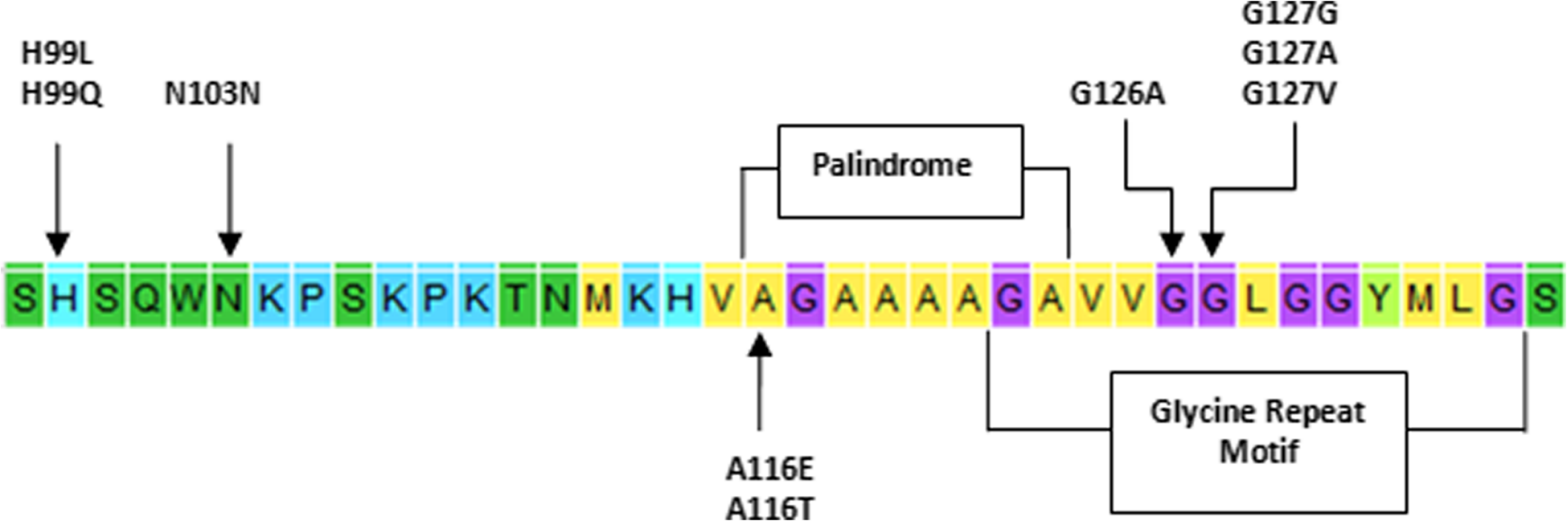
Polymorphisms at cleavage site, palindrome and highly conserved hydrophobic region of PrP

Additional polymorphisms at different positions i.e. G126A (heterozygous and homozygous), G127G (GGG-- > GGT), G127V (heterozygous), G127A (heterozygous), S138S (AGC-- > AGT), I142I (heterozygous), N146S (heterozygous and homozygous), R231R (CGG-- > AGG) and L237L (CTC-- > CTG) were identified Table 1. Most of those polymorphic sites were heterozygous for the specified loci**.** Potential resistant variants M112T, M137T, R151C and N176K were not detected in the studied breeds. Some of the novel variants are localized in the functional domains of prion protein Fig. 1**.**

In the current study, the highly susceptible haplotypes (i.e. haplotypes categorized under groups 4 and 5) were not detected. Instead, ARQ was the dominant allele in the population under study. The haplotype ARR was observed in Menz and Afar breeds at frequencies of 0.02 and 0.05 respectively. ARH/ARH was the most frequent genotype in Menz breeds (0.31). ARQ/ARQ genotype was highly prevalent in Washera (0.68) and Afar (0.78) breeds Table 2. Neither the highly susceptible, VRQ, nor highly resistant, ARR, genotypes were observed in this study. In the present study, no polymorphism was detected at codon 141 L. The population under study was not in Hardy-Weinberg proportion (*χ*_*6*_^*2*^: 35.27 27.58 *p < 0.05*).

**Table 2:** Scrapie associated allele and genotype frequencies of Menz, Washera Afar sheeps’ *PRNP* gene

Allele	Overall Frequency	Menz *N* = 39	Washera *N* = 35	Afar *N* = 23
ARQ	0.62	0.46	0.68	0.78
ARH	0.29	0.41	0.26	0.17
AHQ	0.07	0.11	0.06	–
ARR	0.02	0.02	–	0.05
**Genotype**				
ARQ/ARQ	0.50	0.29	0.62	0.61
ARQ/ARH	0.17	0.17	0.07	0.31
ARH/ARH	0.20	0.31	0.20	–
ARQ/AHQ	0.06	0.14	0.05	–
ARH/AHQ	0.02	0.03	0.03	–
AHQ/AHQ	0.02	0.03	0.03	–
ARQ/ARR	0.02	0.03	–	0.04
ARH/ARR	0.01	–	–	0.04

## Discussion

In the current study, novel amino acid substitutions were detected in the native Ethiopian sheep breed’s *PRNP* gene. These substitutions (H99L, N103 N, A116T, and A116E) are in a proteinase K resistant region of the prion protein. Variants at codon 99 and 103 are localized closer to the peptide cleavage sites. Variants A116T and A116E are placed in the hydrophobic palindrome region (A_116_GAAAAG) which is described as the critical motif in the process of conversion of PrP^C^ to PrP^Sc^ and its propagation [19]. A previous work by Yang et al., 2014 reported PrP with L141R154 haplotype displayed extended beta sheet in the N-terminal palindromic region than the other variants at these codons [3]. The study also predicted possible correlation between conformational change in ovine PrP upon mutation and different forms of scrapie [3]. Hence, the distinctive physicochemical properties of variants might influence the conformational plasticity of prion protein and could potentially trigger the emergence of new scrapie forms.

As in previous studies, non synonymous and synonymous substitutions i.e. G127G, S138S, R231R, L237L, G126A, 126GA, 127GV, 127GA, 142IT, N146S, N146NS were also identified in the population under study [10–12]. In the present study, the variant at codon 127 in particular was highly polymorphic. Amino acids at codon 126 and 127 are localized in the highly conserved glycine rich motif GAVVG_126_G_127_LGGYMLG.This residue was reported to antagonize prion disease development by blocking amyloidal fibril formation [2]. A recent work by Vitale et al.,2019 reported polymorphism at this position and implicated its importance in the normal cellular function of prion protein [20]. The sequence variation in palindrome residue (PrP _113–120_) and glycine repeat regions (PrP _124–128_) synergistically may affect cellular prion protein conformational flexibility.

So far, there are evidences that support the link between scrapie susceptibility, prolonged incubation period and host *PRNP* genotypes. Known polymorphic alleles at codon 136A/V, 154R/H, and 171R/H/Q are implicated in classical scrapie resistance or susceptibility [7]. A recent work by Cassmann et al., 2019 showed 171Q/K genotypes are also associated to scrapie resistance/susceptibility [21]. In atypical scrapie, AHQ, ARQ and ARR genotypes along with homozygosity for phenylalanine at codon 141 are susceptible haplotypes. ARR genotype which is a central protective haplotype in classical scrapie is not protective in atypical scrapie [9, 22].

In the current study, a significant proportion of the population under study were potentially less resistant to classical and atypical scrapie (fall under scrapie resistance category groups 2 and 3). The haplotype, A_136_L_141_R_154_Q_171_, was predominant in the population under study. In the present study, the frequency of the ARQ allele was lower in Menz breed than the other two breeds. On the contrary, ARQ was the most prevalent allele in Afar breed. The observed allele frequency variation might be due to the geographic barriers favoring inbreeding and later results in genetic distinction among breeds.

In countries such as Canada and Czech Republic, where scrapie was once apparently prevalent, the resistant haplotype, ARR, became dominant over the previously reported VRQ and ARQ haplotype [4, 23, 24]. Genetic based eradication program is one of the reasons why ARR haplotype is prevalent in such areas [25, 26]. A study by Migliore S. et al.,2017, speculated positive selection on scrapie resistance goat *PRNP* variants in the places where there was scrapie epidemics [27]. Similarly, in India, China, Israel, Iran, and Turkey where scrapie case has never been epidemic, the predominant allele was ARQ [28–34]. Studies from Tunisia and Algeria reported ARQ, ARR, AHQ, ARH, and VRQ as major alleles. In the same studies, additional polymorphisms which were also previously reported in Spanish and Italian sheep breeds were identified [12, 35–37]. ARQ was recorded at a significantly higher frequency in scrapie-affected Spanish sheep. At the same time, this genotype was dominant in both healthy and scrapie affected sheep breeds [36]. Similarly, ARQ haplotype was predominant in three Sicilian autochthonous sheep breeds. However, ARR haplotye was also reported at high frequency than other common scrapie associated haplotypes [37]. In rare Greek sheep breeds where scrapie has not yet reported, ARQ haplotype was detected in 50% of the sampled population [38]. Despite the long standing selective breeding programs in European countries, there has been no legalized selective breeding practices for scrapie in Bulgaria. A study reported ARQ and ARR as the predominant haplotypes among selected Bulgarian sheep flocks [39]. The high frequency of ARR could be due to the importation of resistant sheep from elsewhere.

Earlier experimental studies linked specific alleles to survival rate after scrapie infection along with the ARQ genotype. T_112_ARQ, AT_137_RQ, AC_151_RQ, and ARQK_176_ haplotypes were identified as potential protective variants [9, 40, 41]. In the present study, these haplotypes were not detected. Rather, the wild type variants i.e. M112, M137, R151, and N176 were highly prevalent. Due to the absence of the resistant genotypes and potential protective alleles such as the aforementioned variants, the population under study was genetically less resistant to scrapie.

In recent years community-based breeding programs have been practiced in Ethiopia [16, 17]. Hence, higher genetic diversity i.e. Heterozygosity and scrapie resistant genotypes would be expected. In the present study however, homozygous genotypes were relatively dominant over heterozygous genotypes. This could be due the limited practice of extensive crossbreeding. Inadequate crossbreeding may compromise efficient animal production [42]. However, genetic diversity in native Ethiopian sheep breed’s *PRNP* was still detected when compared to other breeds worldwide. Therefore, the balance between production improvement through selective breeding and preservation of native breeds should be maintained.

## Conclusion

Two of the novel variants at codon 99 and 103 that are placed closer to the proteinase K cleavage site and the variant at codon 116 that is in the hydrophobic palindrome region along with the variants at codon 127 in glycine repeat domain may influence the conformational flexibility of prion protein and /or introduce a new form of scrapie. Due to the high prevalent group 3 and 4 genotypes and the spontaneity of atypical scrapie, the population under study were less resistant to scrapie. These genetic distributions potentially suggest the need for careful selection during crossbreeding and inbreeding. The results of this study also highlighted the genetic diversity of Ethiopian native sheep breed’s *PRNP* through novel variants and scrapie associated genotype distribution when compared to other breeds worldwide.

## Methods

### Animal selection

According to FAOSTAT 2018, 31,688,157 sheep are kept for production in Ethiopia. Whole blood was taken from 97 genetically unrelated female native sheep (Washera *N* = 39, Menz *N* = 35, and Afar *N* = 23). The sources of the animals for this study were the regional research centres (Andasa Livestock Research Center (ALRC),Melka-Were agricultural research centre and Debre Birhan Agricultural Research Center).

Washera sheep breed is reared in west-east Gojam and AgewAwi zones of Amhara region (Population = 1,227,700) [43]. This breed is commonly known by its short fat tail, short-hair and large body size. Menz sheep breeds are one of the most common sheep breeds in Ethiopia. This breed is distributed in Menz, a zone in North Shewa of Amhara region (Population = 971,400) [43]. Their characteristic feature is a fatty short tail, well developed wooly undercoat with unique spiral horns. Afar sheep, named after the Afar region, are well adapted to harsh environments (Population = 681,900) [43]. These breeds are reared for commercial mutton and wool production. Information on the phenotypes of the breeds was obtained from the Ethiopian Biodiversity booklet, 2018.

### DNA extraction and polymerase chain reaction

Genomic DNA was isolated from the EDTA treated blood using a commercial kit (Geneaid). PCR (Polymerase chain reaction) was carried out to amplify the coding region of the *PRNP* using forward (TCTGCAAGAAGCGACCAAAAC) and reverse (CACAGGAGGGGAAGAAAAGAGG) primers (NM_001009481.1). PCR reaction mixture containing 200 μM of each dNTP, 2 mmol MgCl2, 5 pmol of each primer, 0,05 U Taq polymerase, 10 X PCR buffer (Thermo Fisher Scientific Inc., USA), 10–50 ng of genomic DNA and ddH2O to a final volume of 12,5 ul was used. 2.5 ul of PCR product was used for further analysis.

### Sequencing and bioinformatics

After incubation with 1 U Exo-SAP, a chain termination reaction was performed with BigDye™ terminator v3.1 Cycle Sequencing Kit (Thermo Fisher Scientific Inc., USA). At the final stage, all samples were purified by ethanol precipitation method and sequenced by Applied Biosystems 3500 genetic analyzer (Thermo Fisher Scientific Inc., USA). Chromatograms were checked with FinchTV and aligned using Mega v7.0 software. Hardy-Weinberg equilibrium state for the multilocus allele was calculated using Popgene 32.

## Data Availability

The datasets generated and/or analysed during the current study are available in the [GeneBank] repository, [MN834021-MN834117].

## Abbreviations

*PRNP* gene: Prion protein coding gene
PrP^C^: Cellular prion
PrP^Sc^: Scrapie form of prion
A: Alanine
C: Cysteine
D: Aspartic acid
E: Glutamate
G: Glycine
H: Histidine
I: Isoleucine
K: Lysine
L: Leucine
M: Methionine
N: Asparagine
P: Proline
Q: Glutamine
R: Arginine
S: Serine
T: Threonine
V: Valine
Y: Tyrosine
